# Metabolic tumor volume predicts long-term survival after transplantation for unresectable colorectal liver metastases: 15 years of experience from the SECA study

**DOI:** 10.1007/s12149-022-01796-8

**Published:** 2022-10-14

**Authors:** Harald Grut, Pål-Dag Line, Trygve Syversveen, Svein Dueland

**Affiliations:** 1 Department of Radiology, Vestre Viken Hospital Trust, 3004 Drammen, Norway; 2 Institute of Clinical Medicine, University of Oslo, Oslo, Norway; 3 Department of Transplantation Medicine, Oslo University Hospital, Oslo, Norway; 4 Department of Radiology and Nuclear Medicine, Oslo University Hospital, Oslo, Norway

**Keywords:** ^18^F-FDG PET/CT, Metabolic tumor volume, Colorectal cancer, Liver transplantation, Liver metastases

## Abstract

**Objective:**

To report 15 years of experience with metabolic tumor volume (MTV) of liver metastases from the preoperative ^18^F-FDG PET/CT to predict long-term survival after liver transplantation (LT) for unresectable colorectal liver metastases (CRLM).

**Methods:**

The preoperative ^18^F-FDG PET/CT from all SECA 1 and 2 patients was evaluated. MTV was obtained from all liver metastases. The patients were divided into one group with low MTV (< 70 cm^3^) and one group with high MTV (> 70 cm^3^) based on a receiver operating characteristic analysis. Overall survival (OS), disease-free survival (DFS) and post recurrence survival (PRS) for patients with low versus high MTV were compared using the Kaplan–Meier method and log rank test. Clinopathological features between the two groups were compared by a nonparametric Mann–Whitney *U* test for continuous and Fishers exact test for categorical data.

**Results:**

At total of 40 patients were included. Patients with low MTV had significantly longer OS (*p* < 0.001), DFS (*p* < 0.001) and PRS (*p* = 0.006) compared to patients with high values. The patients with high MTV had higher CEA levels, number of liver metastases, size of the largest liver metastasis, N-stage, number of chemotherapy lines and more frequently progression of disease at LT compared to the patients with low MTV.

**Conclusion:**

MTV of liver metastases is highly predictive of long-term OS, DFS and PRS after LT for unresectable CRLM and should be implemented in risk stratification prior to LT.

## Introduction

Colorectal cancer is one of the most common malignancies and about half of these patients develop liver metastases (CRLM) [1, 2]. Most patients with CRLM have unresectable disease and are treated with palliative chemotherapy with an expected 5-year overall survival (OS) of about 10% [3]. About 20–25% of the patients may receive a curative intended liver resection, but recurrent disease within 3 years after liver resection is seen in a majority and the 5-year OS is about 30–50% in most studies [1, 4].

Liver transplantation (LT) is an established treatment for selected patients with malignancies like hepatocellular carcinoma (HCC) and metastases from low grade neuroendocrine tumors [5–7]. Due to the results from the SEcondary CAncer (SECA) studies at Oslo University Hospital LT is increasingly recognized as a possible treatment option for selected patients with unresectable CRLM. Five-year OS of 60% was reported in the SECA-1 pilot study [8]. Using more strict selection criteria 5-year OS increased to 83% in the SECA-2 study [9]. Lack of liver donors is a challenge worldwide and thus stringent and reliable patient selection criteria of high clinical relevance.

Fluorine-18 fluorodeoxyglucose positron emission tomography in combination with computed tomography (^18^F-FDG PET/CT) is used in cancer detection, staging and response evaluation. ^18^F-FDG PET contributes with functional information beyond conventional anatomical knowledge like size and number of metastases which can be obtained from CT, magnetic resonance imaging and ultrasound. ^18^F - FDG PET/CT is most often used in the setting of recurrent disease and before tentative metastasectomy in CRC [10–12]. In the SECA studies, all patients underwent a whole-body ^18^F - FDG PET/CT prior tentative LT to detect extrahepatic disease which was an exclusion criterion [13].

Metabolic tumor volume (MTV) can be measured and obtained from a ^18^F -FDG PET/CT and represents the active tumor volume in a patient. High MTV has shown to be a surrogate marker of aggressiveness and poor prognosis in several cancer types like lung cancer and oesophageal cancer. In CRLM, the prognostic utility of MTV has been demonstrated both for patients receiving liver resection and palliative chemotherapy [14–16].

The main objective of this retrospective study was to report 15 years of experience with MTV from the preoperative ^18^F-FDG PET/CT to predict long-term survival following LT for unresectable CRLM.

## Methods

### Patient selection

Patients with CRLM from all regions of Norway that may be candidates for LT are referred to the multidisciplinary hepatobiliary meeting at Oslo University Hospital. This meeting consisting of hepatobiliary surgeon, transplant surgeon, radiologist and oncologist decide on treatment options for these patients.

In total, 40 patients were treated with LT for unresectable CRLM in the SECA-1 (*n* = 23) and SECA-2 (*n* = 17) studies in the period November 2006–August 2018. ^18^F-FDG PET/CT scan was a part of the preoperative study protocol to exclude extrahepatic disease [8]. The main inclusion criteria were unresectable colorectal liver only metastases, good performance status (ECOG score 0 or 1), previous chemotherapy and completed radical excision of the primary tumor. Patients who fulfilled all inclusion criteria without evidence of extrahepatic malignant disease on ^18^F-FDG PET/CT and contrast enhanced CT underwent LT. All liver metastases from these ^18^F-FDG PET scans were evaluated in the present study. Chemotherapy was paused the last 4–6 weeks before the ^18^F-FDG PET/CT scan.

The SECA studies were approved by the Regional Ethics Committee and registered at clinicaltrials.gov with registration number NCT01311453 for the SECA-1 study and NCT01479608 for the SECA-2 study. All patients signed an informed written consent.

### ^***18***^***F-FDG PET/CT procedure***

All ^18^F-FDG PET/CT procedures were performed on a hybrid PET/CT system (Siemens Biograph 64 or 16), Siemens Medical Systems, Erlangen, Germany. The patients fasted for minimum six hours and serum glucose level was measured before ^18^F-FDG was injected intravenously. Median injected dose was 383 MBq (range 252–458). Image acquisition started after about 60 min of rest. A whole-body PET from skull base to the upper thigh with an acquisition time of 3 min per bed position and 30% overlap was performed. The PET was reconstructed with 168 × 168 pixels (pixel size 4.06 mm) using OSEM with four iterations and eight subsets (4i/8s) and Gaussian post-reconstruction filter with full-width at half maximum of 5 mm. A low-dose CT without contrast enhancement was used for anatomical information and attenuation correction. The CT acquisition parameters were: 120 kV, 50mAs, and axial slices of 3 mm.

### Image assessments

MTV was obtained from all liver metastases by manually placing a volume of interest (VOI) over each metastasis using a Siemens SyngoVia workstation (version VB10A, Erlangen, Germany) and a fixed threshold of 40%. MTV was only registered if the uptake was higher than the mean liver background uptake × 1.5 + standard deviation of the liver background × 2 [17]. Patients without any metastases with uptake above this value were given an MTV value of zero. Liver background was measured by placing a VOI of 3 cm in the right liver lobe. Total MTV was calculated by adding the values from all metastases for each patient. Maximum standardized uptake value (SUV_max_), SUV_mean_, SUV_peak_, tumor to background (T/B) ratio and total lesion glycolysis (TLG) were also registered. In patients with more than one liver metastasis the highest SUV was registered. SUV_peak_ was defined as the SUV_mean_ of the volume of 1 cm^3^ around the SUV_max_. TLG was calculated by multiplying SUV_mean_ by MTV.

### Statistical analysis

Statistical analyses were performed with SPSS (IBM, version 27, Chicago, Illinois, USA). A receiver operating characteristic (ROC) analysis was used to determine an MTV cut-off value for predicting OS. Cut-off values for SUV_max_, SUV_mean_, SUV_peak_, T/B ratio and TLG were also obtained. OS was defined as time from LT until death or end of follow-up 22nd of December 2021. Disease-free survival (DFS) was defined as time from LT to the detection of suspected metastases or local recurrence on either CT, MRI or PET/CT. Post recurrence survival (PRS) is OS minus DFS. Survival curves were generated using the Kaplan–Meier method and the groups were compared using the log rank test. Synchronous liver metastases were defined as liver metastases occurring less than 12 months and metachronous more than 12 months after the CRC diagnosis. The response evaluation criteria in solid tumors (RECIST) on CT were used to evaluate response to chemotherapy prior to LT. Progression of disease was defined as a 20% or greater increase in the sum of the longest diameter of the target lesions. An increase in carcinoembryonic antigen (CEA) level prior to LT was also interpreted at progression of disease. A nonparametric Mann–Whitney *U* test was used for comparing continuous data and Fishers exact test was used for comparing categorical data. A 2-tailed probability level less than 0.05 was considered statistically significant.

## Results

### Patient and baseline characteristics

Patient and baseline characteristics for all patients and a comparison of the MTV low (< 70 cm^3^) versus MTV high (> 70 cm^3^) group is given in Table 1. The patients with high MTV had significantly higher CEA levels, number of liver metastases, size of the largest liver metastasis, N-stage, number of chemotherapy lines and frequency of progressive disease at LT compared to the patients with low MTV. A comparison of clinopathological features and PET metrics is given in Table 2.

**Table 1:** Patients and baseline characteristics

	All	MTV low	MTV high	*p* value^+^
Patients, *n*	40	26	14	
Age at LT, median (range)	57 (34–71)	58 (34–71)	55 (44–64)	0.143
Gender*				
Male	22 (55)	14 (54)	8 (57)	1.000
Female	18 (45)	12 (46)	6 (43)	
Primary tumor*				
Colon	26 (65)	18 (69)	8 (57)	0.501
Rectum	14 (35)	8 (31)	6 (43)	
T-stage primary tumor*				
T0	2 (5)	0	2 (14)	0.417
T1	1 (2)	1 (4)	0	
T2	4 (10)	3 (11)	1 (7)	
T3	32 (80)	21 (81)	11 (79)	
T4	1 (2)	1 (4)	0	
N-stage primary				
N0	18 (55)	15 (58)	3 (21)	0.072
N1	14 (35)	8 (31)	6 (43)	
N2	8 (20)	3 (11)	5 (36)	
Time of liver metastases*				
Synchronous	35 (88)	21 (81)	14 (100)	0.143
Metachronous	5 (12)	5 (19)	0	
Chemotherapy before LT*				
1 line	19 (48)	18 (69)	1 (7)	< 0.001
2 lines	17 (42)	8 (31)	9 (64)	
3 lines	4 (10)	0	4 (29)	
Progressive disease at LT*				
Yes	16 (40)	5 (19)	11 (79)	< 0.001
No	24 (60)	21 (81)	3 (21)	

*LT* liver transplantation, *MTV* metabolic tumor volume

^*^n (%)

^+^Patients with low MTV compared to patients with high MTV. Nonparametric Mann–Whitney *U* test for continuous data and Fishers exact test for categorical data

**Table 2:** Results

	MTV low	MTV high	*p* value^+^
Patients, *n*	26	14	
Time from diagnosis to LT, months*	22 (6–112)	22 (6–36)	0.834
Time from primary surgery to LT, months*	17 (2–111)	21 (6–36)	0.989
Time from PET/CT to LT, days*	103 (12–258)	63 (17–293)	0.424
CEA level, μg/L*	3 (1–33)	95 (2–2002)	< 0.001
Number of liver metastases (CT), n*	6 (1–40)	11 (4–53)	0.015
Largest liver metastasis (CT), mm*	29 (3–52)	83 (13–130)	< 0.001
Total lesion glycolysis*	15.63 (0–211.26)	1144 (266–4438)	< 0.001
SUV_max_*	4.35 (2.33–13.05)	12.6 (6.31–21.45)	0.001
SUV_peak_*	3.48 (1.92–10.86)	9.85 (3.21–17.48)	< 0.001
SUV_mean_*	3.04 (1.62–7.96)	6.48 (2.22–13.27)	0.001
Tumor to background ratio*	2.27 (1.00–6.13)	6.01 (1.95–11.08)	< 0.001
Fong clinical risk score*	2 (1–4)	4 (3–5)	< 0.001
Tumor burden score*	8.5 (4.2–50.3)	13.6 (7.1–53.1)	< 0.010
First site recurrence, *n*	Lung (11), Liver (1), lymph node (2), multiple (1), local (1), other (3)	Lung (6), Liver (2), lymph node (2), multiple (4), local (0), other (0)	0.233

Low versus High Metabolic Tumor Volume (MTV)

*MTV* metabolic tumor volume, *LT* liver transplantation, *CEA* carcinoembryonic antigen, *SUV* standardized uptake value

*Median (range)

^+^Nonparametric Mann–Whitney *U* test for continuous data and Fishers exact test for categorical data

### PET measurements

The ROC analysis determined an MTV cut-off value of 66.09 cm^3^. Twenty-six patients had low MTV (< 70 cm^3^) and 14 patients had high MTV (> 70 cm^3^). Cut-off values based on the ROC analysis were obtained for SUV_max_ (5.87), SUVmean (3.54), SUV_peak_ (5.49), T/B-ratio (5.28) and TLG (238.80). The MTV low group had significantly lower SUV_max_, SUV_mean_, SUV_peak_, T/B-ratio and TLG compared to the MTV high group (Table 2).

### Overall survival analysis

**Fig. 1:**
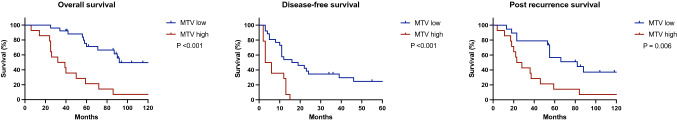
Kaplan–Meier overall, disease-free and post recurrence survival curve for all SECA patients (*n* = 40). Patients with low MTV (< 70 cm^3^, blue line) had significantly improved survival compared to patients with high MTV (> 70 cm^3^, red line). *MTV* metabolic tumor volume

OS Kaplan–Meier survival curve for patients with low (*n* = 26) versus high (*n* = 14) MTV is shown in Fig. 1. OS at 5 and 10 years were 76 and 50% in the MTV low patients compared to 21 and 7% in the patients with high MTV (*p* < 0.001).

**Fig. 2:**
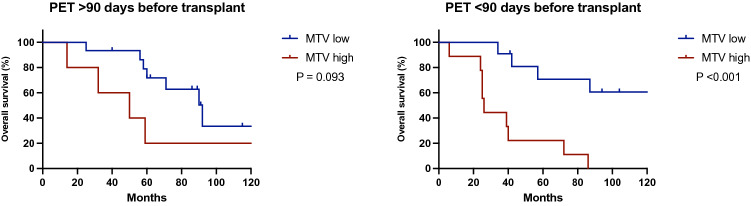
Kaplan–Meier overall survival curves for SECA patients who underwent ^18^F-FDG PET/CT > 90 days (*n* = 20) and < 90 days (*n* = 20) before LT. Overall survival was significantly improved (*p* < 0.001) in patients with low MTV (< 70 cm^3^, blue line) compared to patients with high MTV (> 70 cm^3^, red line) when the ^18^F-FDG PET/CT was performed < 90 days before LT but not when the ^18^F-FDG PET/CT was performed > 90 days before LT. *LT* liver transplantation, *MTV* metabolic tumor volume

In patients where the ^18^F-FDG PET/CT was performed more than 90 days before LT (*n* = 20), the effect of low MTV was not significant compared to high MTV (*p* = 0.093, Fig. 2). Twenty patients underwent ^18^F-FDG PET/CT less than 90 days before LT. In this subgroup, 71 and 61% were alive at 5 and 10 years when MTV was low (*n* = 11), and 22% and 0% MTV was high (*p* < 0.001, Fig. 2).

**Fig. 3:**
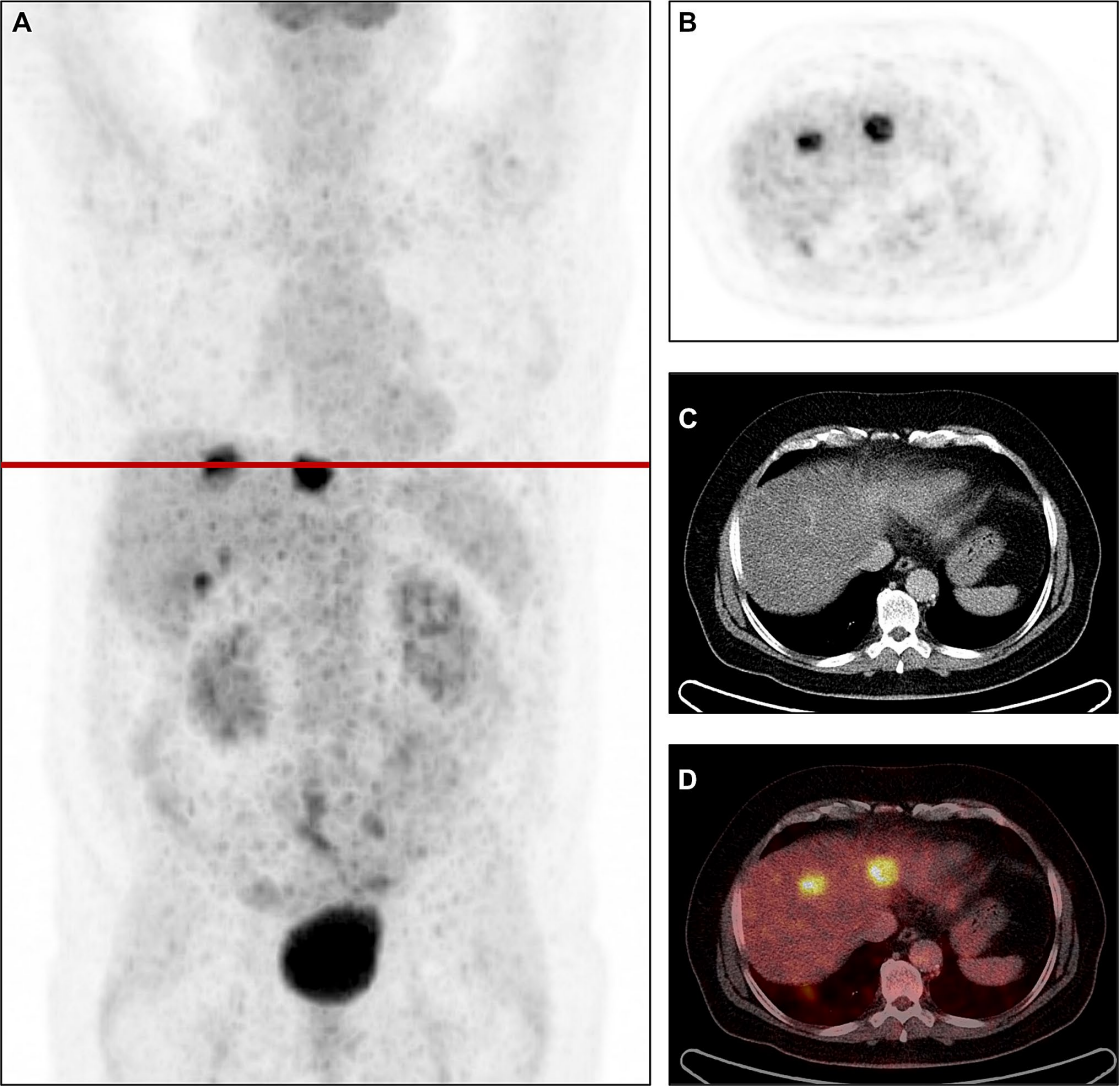
**A** Maximum intensity projection, **B–D** ^18^F-FDG PET, CT and fused ^18^F-FDG PET/CT of a patient with low MTV (41.10 cm^3^). The patient is still alive almost 14 years after LT despite pulmonary relapse. *LT* liver transplantation, *MTV*  metabolic tumor volume

**Fig. 4:**
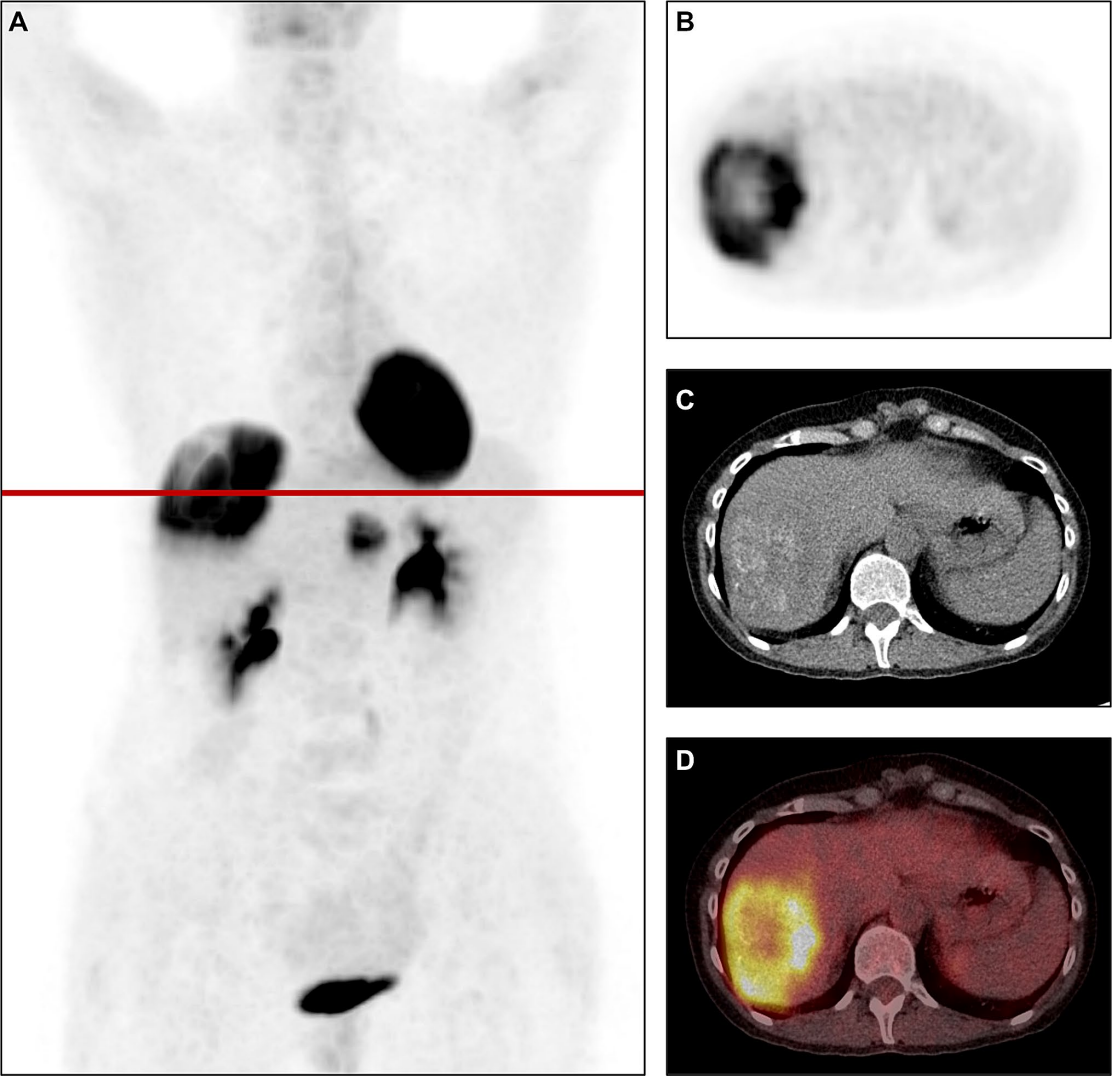
**A** Maximum intensity projection, **B–D** ^18^F-FDG PET, CT and fused ^18^F-FDG PET/CT of a patient with high MTV (194.35 cm^3^). The patient developed a multiple site recurrence 3 months after and died only 14 months after LT. *LT *liver transplantation, *MTV* metabolic tumor volume

TLG analysis stratified the exact same patients and gave the same survival results as MTV. SUV_max_, SUV_mean_, SUV_peak_ and T/B-ratio showed non-significant differences when comparing OS for patient with low versus high values (*p* = 0.401, 0.366, 0.355 and 0.056). Figure 3 illustrates one patient with low MTV still alive almost 14 years after LT despite pulmonary relapse. Figure 4 illustrates one patient with high MTV who developed a multiple site recurrence and died only 14 months after LT.

### Disease-free and post recurrence survival analysis

DFS and PRS estimated survival analysis for MTV low versus MTV high is shown in Fig. 2. All 14 patients with high MTV and 19 out of 26 with low MTV had developed recurrence. DFS at 1,3 and 5 years was 53, 31 and 21% in the MTV low group compared to 36, 0 and 0% in the MTV high group (*p* < 0.001, Fig. 1). Median DFS was 16 months in the MTV low group compared to 4 months in the MTV high group (*p* < 0.001, Fig. 1). Although the MTV low patients had somewhat higher number of patients with lungs as first site recurrence compared to the MTV high patients, no significant difference in first site recurrence was observed (*p* = 0.233, Table 2).

PRS was significantly improved in the MTV low compared to the MTV high group with 57 and 37% alive at 5 and 10 years compared to 14 and 7% (*p* = 0.006, Fig. 1).

## Discussion

The main finding in this study is that MTV of liver metastases obtained from the preoperative ^18^F-FDG PET/CT is highly predictive of long-term OS, DFS and PRS following LT for unresectable CRLM. The result is in line with the first report from the SECA-1 pilot study where MTV was significantly associated with improved 5-year OS (*p* = 0.026) [18]. In the present study where data from the SECA-1 and 2 studies were pooled and the patients were observed for a longer period of time the ability of MTV to predict long-term survival was stronger than the first report underlining the consistent predictive properties of MTV. Based on these results, we recommend that assessment of MTV should be obtained for all patients considered for LT for CRLM as a mandatory part of the LT workup. MTV is quite easy to obtain and can be used in risk stratification in conjunction with other biomarkers of prognosis. Because TLG stratified the exact same patients as MTV and SUVs and T/B-ratio showed non-significant differences when comparing OS, it seems like obtaining only MTV is sufficient for clinical use.

In the SECA studies most patients had recurrent disease [9]. In the present study, DFS was also significantly improved in the group with low MTV. However, previous reports have shown that patient with pulmonary relapse available for surgical resection have a long survival and patients with extrapulmonary recurrence have poor survival [8, 9, 19]. Hence, the site of recurrence is more important than the explicit DFS and there is a poor correlation between DFS and OS, suggesting that DFS is of limited value as a parameter of treatment efficacy in LT for CRLM. This diverges from LT for HCC where most patients do not recur but those with recurrence have poor survival in general [20, 21].

We have previously shown that ^18^F-FDG PET/CT is an important pretransplant examination to detect excluding extrahepatic disease prior to LT [13]. The subgroup analysis suggests that the ^18^F-FDG PET/CT should be performed < 90 days prior to LT (Fig. 2). This underlines the dynamics of the disease, suggesting that repeated investigations may be advised in situations with long transplant waiting times. We therefor recommend repeated ^18^F-FDG PET/CT if time from last scan exceeds 3 months in patients on the waiting list or during workup for possible LT for CRLM. This is also in line with most follow-up programs for cancer patients where imaging usually is performed every 3 months.

MTV is reflecting the metabolically active tumor load and it is plausible that the risk of tumor growth and metastatic potential is linked to active liver tumor load prior to LT. This could explain the improved OS and DFS in the MTV low patients. Despite that all SECA patient undergo a contrast enhanced diagnostic CT, MRI of the liver and a whole body ^18^F-FDG PET/CT to exclude extrahepatic disease, very small lesions or microscopic residual disease are not detectable by any imaging modality. The high recurrence rate in the SECA studies underlines the importance of close surveillance following LT to tailor treatment of recurrence. In the SECA-1 study where patients with progression of CRLM during chemotherapy also were considered for LT the ^18^F-FDG PET/CT performed before tentative LT detected extrahepatic disease in about 1/3 of the patients who were excluded [13].

Although not significant, the MTV low group had more patients with the lungs as first site of relapse (Table 2). Many of these patients were underwent pulmonary resection. Previous publications have shown that patients with pulmonary relapse have favorable outcome compared to patients with another first site of relapse [19, 22], and this is the most plausible explanation for the increased PRS in the patients with low MTV.

All SECA patients had unresectable CRLM and had received chemotherapy before ^18^F-FDG PET/CT. Thus, the MTV would be a surrogate marker of tumor biology with low MTV as a marker of less aggressive disease because of the response on chemotherapy. The group with high MTV had significantly more patients with progression of disease at LT compared to the group with low MTV (79% vs. 19%) and significantly higher number of patients with 2 or 3 lines of chemotherapy (93% vs. 31%) suggestive of more aggressive disease (Table 1). In line with our experience, several studies have shown that ^18^F-FDG PET parameters can predict treatment response and outcome following neoadjuvant chemotherapy in locally advanced rectal cancer and response of colorectal cancer metastases after chemotherapy which also is correlated to outcome [23].

To our knowledge, no other studies have reported the prognostic properties of MTV for patients undergoing LT for CRLM. However, MTV from the pre-treatment ^18^F-FDG PET/CT has shown to be prognostic in patients treated with selective internal radiation therapy (SIRT) with yttrium-90 for unresectable CRLM and resection for CRLM [24–27]. Several studies have shown that MTV derived from ^18^F-FDG PET/CT can predict recurrence and survival after LT for HCC [28, 29].

Our study has limitations. It was retrospective and the total number of patients is relatively low (*n* = 40). However, this is the by far largest material on LT for CRLM worldwide and the observation time is very long. Also, our study showed highly significant differences in survival. In general, variability in the time interval between ^18^F-FDG PET/CT and LT could represent a source of possible bias. Because no difference in time between the ^18^F-FDG PET/CT and LT were observed when comparing the MTV low to the MTV high group this was probably not critical in the present study. The ^18^F-FDG PET performed in the SECA studies followed a standard clinical protocol and did not include respiration gating for the imaging of liver metastases or for the detection of extra hepatic lesions in the liver hilum. Although most inclusion and exclusion criteria in the SECA-1 and SECA-2 studies were the same no patients with progression of disease or less than 1 year from the CRC diagnosis were eligible for inclusion in the SECA-2 study. Thus, the SECA-2 patients could have had more favorable tumor biology compared to SECA-1. OS at 5-years in the SECA-1 study was 60 compared to 83% in SECA-2. However, in the present study, the patients from the SECA-1 and SECA-2 studies were pooled and not compared.

## Conclusion

MTV of liver metastases is highly predictive of long-term OS, DFS and PRS after LT for unresectable CRLM and should be implemented in risk stratification prior to LT.
